# The Role of Goal Source in Escalation of Commitment

**DOI:** 10.1027/1618-3169/a000627

**Published:** 2024-12-12

**Authors:** Jong Seok Lee, Mark Keil, Kin Fai Ellick Wong, Hyung Koo Lee

**Affiliations:** ^1^ Department of Accounting & Information Management, The University of Tennessee, Knoxville, TN, USA; ^2^ Department of Computer Information Systems, Georgia State University, Atlanta, GA, USA; ^3^ Department of Management, Hong Kong University of Science & Technology, Kowloon, Hong Kong SAR; ^4^ Department of Information Technologies, HEC Montreal, Montreal, QC, Canada

**Keywords:** escalation of commitment, goal setting theory, goal source, inherited goal

## Abstract

**Abstract:** Escalation of commitment is an important decision problem that occurs across different decision contexts. Recognizing that escalation involves one’s effort to achieve some form of a goal, researchers have attempted to understand escalation of commitment as a goal-pursuing activity. Prior research works have suggested that escalation situations consist of (1) an initial goal setting phase and (2) an escalation decision-making phase and have investigated how goal difficulty and goal specificity influence escalation decisions. However, they have neglected the potential role of the goal source in escalation situations. In this study, we aim to advance our understanding of escalation of commitment by examining the relationship between goal source and escalation. Specifically, by drawing on distinct characteristics of escalation situations, we conceptualize a new form of goal source, namely inherited goals, and examine its effect on escalation of commitment compared with self-set and assigned goals that are well-known goal sources in goal-setting theory (GST). We conducted two laboratory experiments and found evidence suggesting that individuals who had inherited goals (i.e., those who did not take part in initial goal setting and did not invest effort in pursuing the previous course of action) are less likely to fall into the escalation trap.


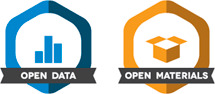


One of the challenges that individuals often face is deciding whether to abandon a previously chosen course of action that has not produced the desired outcome. Individuals frequently become overcommitted to a failing course of action, a phenomenon known as “escalation of commitment” (Staw, 1976). Escalation of commitment has been observed across a wide variety of contexts, such as bank loans (Whyte et al., 1997), investment decisions (Karlsson et al., 2005), and hiring and promotion decisions (Wong et al., 2006).^1^

One prominent characteristic of escalation situations is that individuals begin with an initial decision to embark on a particular course of action (a means) with the aim of achieving a goal (an end), and this initial decision is followed by a subsequent escalation decision concerning whether to recommit to the previously chosen course of action that has not led to the desired outcome (Brockner, 1992). Hence, research suggests that escalation of commitment can be understood as a goal-pursuing activity (e.g., Fox & Hoffman, 2002). Recognizing that goals at the outset of a course of action can influence the subsequent escalation decision, researchers have examined the relationships between initial goal characteristics, such as goal difficulty (Lee et al., 2015) and goal specificity (Lee et al., 2012), and escalation. Despite the contributions of these escalation studies from a goal-setting theory (GST) perspective, their focus is limited to the difficulty and specificity of initial goals. Extant literature neglects the role of goal source in escalation of commitment. This is an important gap because when a previously chosen course of action does not lead to goal achievement, leadership changes can occur, and incoming managers must decide whether to continue the previously chosen course of action to achieve the same goal. The goal in such situations can be said to be *inherited* in the sense that it is passed on to someone who inherits a project or initiative undertaken by a predecessor. Therefore, it is this escalation context that gives rise to a new form of goal source that has not been discussed or conceptualized in the GST literature: namely, inherited goals. We believe a major contribution of our research is to conceptualize inherited goals by marrying GST with escalation and empirically showing that in escalation situations inherited goals have an effect that can be differentiated from self-set goals.

## Escalation of Commitment

To date, escalation of commitment has been studied from a variety of perspectives, including self-justification theory (Staw, 1976), prospect theory (Whyte, 1993), approach avoidance theory (Brockner, 1992), and negative affect aversion (Wong et al., 2006). While advancing our understanding on escalation, these perspectives tend to neglect that the initial decision to embark on a course of action begins with a “goal intention” phase, which involves setting a goal to be achieved.

More recently, a few studies have examined the connection between the initial goal-setting phase and the escalation decision, which occurs at a later point in time after negative feedback is received. Lee et al. (2012) found that extremely difficult goals or specific goals that were made at the outset of a new software development project tended to reduce decision makers’ willingness to continue a troubled project. Another study examined the underlying mechanism behind goal difficulty and escalation in a series of laboratory experiments (Lee et al., 2015) and found that goal valence (i.e., the anticipated satisfaction of attaining a goal) and goal expectancy (i.e., the perceived likelihood that allocating additional resources will lead to goal attainment) mediated the relationship between goal difficulty and escalation. Moreover, the relationship between goal difficulty and escalation of commitment followed an inverted U-shape. That is, while more difficult goals tend to lead to greater escalation, this relationship changes when the goal is perceived as impossible to attain. In such cases, more difficult goals do not increase escalation and may even reduce it. In this study, we extend this line of work by exploring the role of goal source (Locke & Latham, 2002) in escalation of commitment.

## Goal Setting Theory and Goal Source

Goal-setting theory (GST) suggests that the effect of a goal on performance can differ depending on various factors such as goal difficulty (Latham et al., 1982), goal orientation (Seijts et al., 2004), performance feedback (Cianci et al., 2010; Kuvaas, 2011), or the number of goals (Emsley, 2003). Of interest to this study is the goal source, which reflects the level of one’s influence on setting a goal (Locke & Latham, 2006). For example, one may have complete control over setting a goal (self-set goal) or may have no control, instead receiving a goal that is set by others (assigned goal). Early GST research suggested that self-set goals are more effective in improving task performance. Erez et al. (1990) found that for moderate and difficult goals, individuals with self-set goals exhibited better task performance compared to those with assigned goals. However, Locke and Latham (2012, p. 10) conclude that “by 1990, it was clear that … self-set goals are as effective, but not more effective in bringing about goal commitment and increasing performance than an assigned or a participatively set goal.” This is because when there is a logic or rationale offered, goal commitment or performance tend not to suffer when assigned goals are utilized (Latham et al., 1988). Despite the advances made in our understanding of goal source by prior GST research, inherited goals, which may result from a unique context in which a predecessor has failed to achieve a goal, have not been conceptualized or empirically examined in GST.

## Goal Source and Escalation of Commitment

Typically, goal setting involves two actions: (1) setting a goal (individually or participatively)^2^ or being assigned a goal and (2) working on a chosen course of action with the aim of attaining the goal. However, escalation situations typically involve an additional action, that is, deciding whether to continue a failing course of action (Staw, 1982). In escalation situations, we suggest that one’s involvement can differ depending on each of these three actions. Individuals with a self-set goal are involved in all three actions, whereas individuals with an assigned goal are typically involved in the latter two only. In addition to these two variations, an individual may be asked to take part only in deciding whether to continue or not. We call this type of goal source an inherited goal (Table 1). The conceptualization of inherited goals is important in the context of escalation, as escalation situations have an additional decision point where the decision maker must decide whether to continue pursuing the goal.

**Table 1:** Three types of goal source in escalation situations

Type of goal source	Involvement in initial goal setting	Involvement in pursuing previous course of action	Involvement in decision to continue
Self-set goal	Yes	Yes	Yes
Assigned goal	No	Yes	Yes
Inherited goal	No	No	Yes

These three distinct types of goal source allow us to theorize about the relationship between goal source and escalation of commitment. Involvement in goal setting (i.e., self-set goals) may cause individuals to put in a greater amount of effort toward achieving the goal and, thus, can result in better task performance than no involvement in goal setting (i.e., assigned goals; Erez et al., 1985). This assumes, however, that no logic or rationale is provided for assigned goals. We therefore expect that individuals with self-set goals will be more likely to continue a previously course of action that did not lead to goal achievement as compared to individuals with assigned goals. Furthermore, we posit that a goal that was set *and* failed to be attained by a predecessor (i.e., inherited goal) is even less likely to be accepted by an individual, compared with either a self-set goal or an assigned goal that failed to be attained because individuals with an inherited goal are *neither* involved in initial goal setting *nor* in pursuing the previous course of action. Such individuals are therefore less likely to continue pursuing a previously chosen course of action toward achieving the failed goal because they are less motivated to attain the goal. In sum, we posit that escalation of commitment associated with self-set goals is reduced when goals are inherited, with assigned goals generating an intermediate amount of escalation. Thus, we propose the following hypothesis:

*Hypothesis 1 (H1)*: Individuals’ willingness to continue a failing course of action will be reduced as the goal source changes from self-set goal to assigned goal to inherited goal.

## Methods

In this study, we conducted two laboratory experiments. Experiment 1 was conducted to test the relationship between goal source and escalation. In Experiment 2, we refined our experimental design to rule out a potential rival explanation and to replicate the results of Experiment 1, thereby demonstrating the robustness of our findings (Lykken, 1968).

## Experiment 1

### Participants and Experimental Design

We recruited 99 undergraduate students enrolled in an introductory business course at a large university in the southeastern US (*M*_age_ = 22.31, *SD* = 6.14; 54% male, 46% female). Participants were randomly assigned to one of three treatment groups: self-set goal, assigned goal, and inherited goal (conceptualized as a situation in which there was no participation in either goal setting or the previous course of action that did not lead to goal attainment as per Table 1). We did not include a control group because (1) it is not obvious what an appropriate control condition would be in our research context and (2) our objective is to compare the effects of different treatments (self-set, assigned, and inherited goals) on escalation.

The goal source was manipulated using a yoked design in which a pair of participants receive exactly the same treatment, but only one person in the pair is given an opportunity to exert control over the treatment (e.g., setting a goal; Tybout et al., 2001). This approach has the advantage of ensuring that a pair of participants receive identical experience (in this case with respect to goal difficulty) and has also been used in the escalation literature (Wong et al., 2006). For this study, we only examined difficult goals to maximize the effect of goal difficulty (Erez et al., 1990). The difficult goals chosen by participants in the self-set goal group were assigned to participants in the assigned goal group. The goals and performance of the participants in the self-set goal group were assigned to participants in the inherited goal group. This ensured that each participant in the assigned and the inherited goal groups was working with the identical goal as the individual that s/he was yoked to in the self-set goal group. This yoked design allowed us to create a triad of three participants sharing the same goal (a participant in the self-set goal condition, the assigned goal condition, and the inherited goal condition).

### Decision Task, Procedure, and Measures

We adopted a letter counting task from prior escalation research (Lee et al., 2015). At the beginning of the experiment, participants in all three experimental groups were given instructions pertaining to the task, goal, and reward rules, and then were asked to set a goal, were assigned a goal, or told they had inherited a goal from a predecessor. In the self-set goal condition, participants were given instructions that contained an anchor and asked to set a goal as follows: “Set a goal for the percentage of ‘a’ letters in the passage that you think you can identify (by circling) in 1 min, for example 100%”. Participants then recorded their goal as follows by filling in the blank: “My goal is to be able to identify and circle ____% of the “a” letters in 1 min.” Participants in the assigned goal group were then yoked to a participant in the self-set goal group. Participants in the inherited goal group yoked in the same fashion and were informed that they had been brought in to replace another individual who had previously set a goal but failed to attain it. In sum, triads of three participants were yoked based on a goal set by a participant in the self-set goal group.

Participants were informed that: (1) they would be rewarded based on their performance, (2) the reward would be in direct proportion to the goal, provided that the goal was attained, and (3) there would be no additional reward for exceeding the goal. Rewards were set at the rate of 10 cents for every percent of a’s identified in the passage of text. Before performing the task, participants answered two questions (each on a 7-point rating scale) pertaining to task specific self-efficacy that were adapted from Whyte et al. (1997): (1) How would you describe your capability in identifying and circling “a” letters? And (2) how confident are you in your ability to meet a challenging goal with respect to identifying and circling “a” letters within a prescribed period of time?

Participants in the self-set and assigned goal groups only performed a task that involved identifying the letter “a” by circling each occurrence in a short text passage. Each participant was instructed to spend one minute circling a’s, and that time would be given afterward to count and record the number of a’s they circled. The passage of text had 291 words and contained 110 a’s (ESM 1). The percentage of a’s that a participant set out to identify in the article constituted the goal.

After performing the task, participants counted and recorded the number of a’s they identified, whereupon the administrator provided individual feedback on their performance. The administrator visually compared each individual’s goal with the reported count to see whether the goal was attained. Participants who did not attain their goal were given this as feedback along with the exact percentage of a’s in the passage they identified as a measure of their performance. Participants who did not attain the goal were invited to continue with the experiment. Participants who attained their goal were given a monetary reward and dismissed from the experiment. Participants in the inherited goal group received the information on the predecessor’s performance.

Next, participants answered two questions pertaining to perceived goal difficulty on a 7-point rating scale: (1) based on the feedback received after Phase 1, I believe that the goal was difficult to achieve and (2) based on the feedback received after Phase 1, meeting the goal was difficult. Then, participants were informed that they would be given 2 more min and were asked to indicate what proportion of the 2 min they would choose to allocate between two tasks. The first task was to continue identifying a’s with the aim of meeting the original goal. Participants were informed that if they chose this task, the progress they made in the first trial (Phase 1) would be accumulated in the second trial (Phase 2). The second task involved setting a new goal representing the percent of i's that they would try to identify in the same text (any goal between 1% and 100% could be chosen) and working to attain the new goal. The rewards followed the same rules as in Phase 1 for meeting either the original goal or their new goal. After allocating the time they planned to spend between the two tasks, however, participants did not actually work on either task.

**Figure 1:**
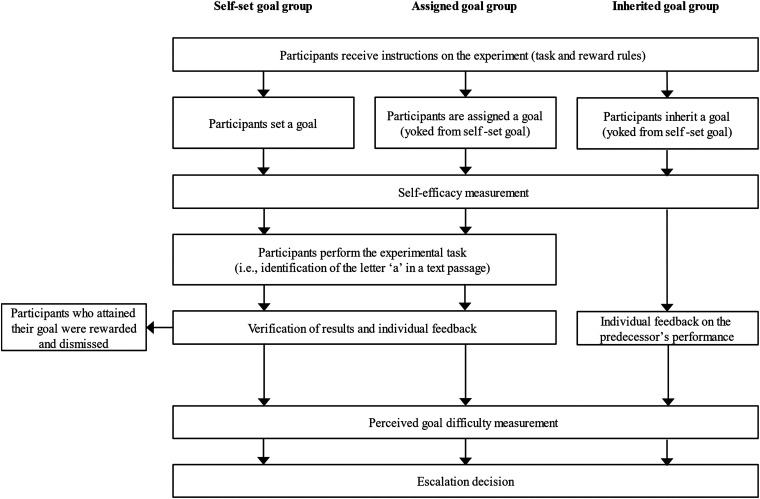
Experimental procedure (Experiment 1).

Overall, the experimental task and procedure were designed to be consistent with the escalation literature and thus incorporated the following three characteristics: (a) embarking on a course of action with some goal in mind; (b) negative feedback about failure to attain the goal; and (c) an opportunity to invest additional resources to pursue a previous course of action (Staw, 1982). A flowchart depicting the experimental procedure is shown in Figure 1.

The dependent variable in our experiment was the percentage of time allocated in Phase 2 to the original task, similar to the allocation of resources used in prior escalation studies (e.g., Conlon & Garland, 1993; Staw, 1976).

### Results

**Figure 2:**
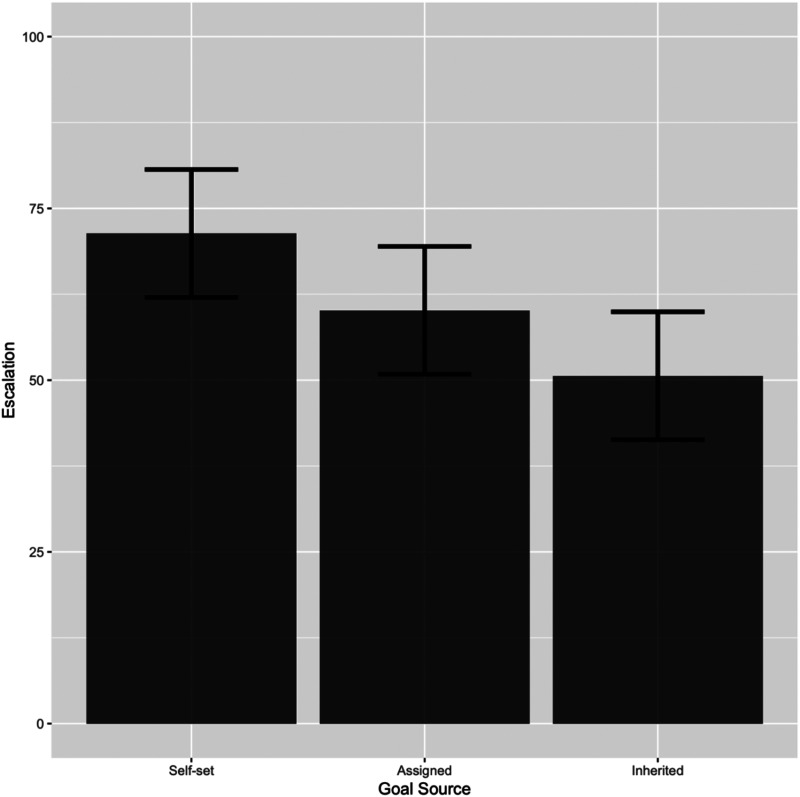
95% CIs of escalation decision (Experiment 1).

A triad of three participants sharing the same goal (a participant in the self-set goal condition, the assigned goal condition, and the inherited goal condition) became our unit of analysis. A total of four participants were excluded from further analysis because they attained their goals in Phase 1. Only those triads in which neither the participants in the self-set goal group nor those in the assigned goal group attained the goal were included in the analysis. On this basis, 31 triads were retained for analysis. The mean goal set by the participants in the self-set goal group was 74.00 (*M* = 74.00, *SD* = 19.88). Because of the yoked design, the goals were the same in all three treatment groups. Once again, our dependent variable of interest was the mean percentage of time allocated to the original task. Participants in the self-set goal group allocated on average 71% of the 2 min to the original task (*M* = 71.35, *SD* = 29.44), participants in the assigned goal group allocated on average 60% of the 2 min to the original task (*M* = 60.16, *SD* = 32.85), and participants in the inherited goal group allocated on average 51% of the 2 min to the original task (*M* = 50.65, *SD* = 14.76). The pattern of results obtained was consistent with our hypothesis, suggesting that escalation of commitment associated with self-set goals is reduced when goals are inherited, with assigned goals generating an intermediate amount of escalation. 95% Confidence intervals (CI) calculated based on repeated-measure error terms (Loftus & Masson, 1994) for each experimental group are shown in Figure 2.

The effect of goal source was tested statistically through repeated measures regression following the procedure delineated in Cohen and Cohen (1983), which focuses on testing the significance of incremental variance accounted by a repeated-measures factor using an *F*-statistic. We chose this analysis because our experiment involved a yoked design in which three individuals constituted a triad (self-set, assigned, and inherited). This enabled repeated measure comparisons among goal source conditions with respect to escalation while controlling for individual differences in a triad. We followed a least-squares dummy variable approach (Sayrs, 1989) in which *N* − 1 dummy-coded subject vectors were created (representing each of the participants) and entered in the first step of the regression to control for individual differences in a triad. The least-squares dummy variable approach helps “remove between-subjects variance prior to examination of the variables of interest” (Donovan & Williams, 2003, p. 384). First, in Model 1, the dummy vectors were entered into the regression to predict the mean percentage of time allocated to the original task (DV). Second, in Model 2, self-efficacy was entered into the regression to control for between-subject differences in self-efficacy. Phase 1 performance was not included in this step as the participants in the inherited goal condition did not engage in Phase 1. Third, in Model 3, the goal source was entered into the regression. The *F*-change in Model 3 was found to be significant (∆*F* = 5.06, *p* < .01), indicating that the goal source had an effect on escalation controlling for both individual differences in a triad and self-efficacy.

Next, we conducted a between-subjects ANCOVA with a post hoc analysis (Tukey HSD) to pinpoint significant differences between the three goal source groups. Self-efficacy was included as a covariate. We found that the mean percentage of time allocated to the original task was significantly higher in the self-set goal group than in the inherited goal group (*p* < .01). We did not find a statistically significant difference in the other pairwise comparisons. Nonetheless, the pattern of results obtained (see Figure 2) suggests that escalation is reduced as the goal source changes from self-set goals to assigned goals and then to inherited goals. This provides some novel insights into the relationship between different goal sources and escalation of commitment.

## Experiment 2

The objective of Experiment 2 was to address a potential limitation of Experiment 1, which did not include what might be a key factor influencing the causal relationship between goal source and escalation, namely, personal responsibility (Staw, 1976). One could argue that the effect observed in Experiment 1 was because participants in the self-set goal group felt greater responsibility for the initial goal than did participants in the inherited goal group. To rule out this rival explanation, we wanted to control for perceived personal responsibility.

While prior research on escalation highlights the importance of personal responsibility, self-set goals and personal responsibility are different and they may influence escalation through different mechanisms. Specifically, when an individual is personally responsible for initiating a course of action and receives negative feedback, s/he may try to justify the negative outcomes and engage in escalation to reduce cognitive dissonance (Staw, 1976). On the other hand, GST suggests that self-set goals increase one’s effort toward goal attainment (Erez et al., 1990), which can lead to escalation. Thus, while self-set goals can heighten one’s sense of personal responsibility, the two constructs are distinct and the mechanisms through which they influence escalation may be different.

A secondary objective of Experiment 2 was to determine if the findings of Experiment 1 in which self-set goals led to a greater investment of time to the original task compared to inherited goals could be replicated using a longer decision task and a different measure of escalation.

### Participants, Experimental Design, and Procedure

Participants in Experiment 2 were 109 undergraduate students from the same business course as in Experiment 1 (*M*_age_ = 21.88, *SD* = 4.76; 53% male, 47% female), but they were recruited from different sections, thus, ensuring that no participant from Experiment 1 took part in Experiment 2. Like Experiment 1, the goal source was manipulated using a yoked design: self-set, assigned, and inherited goal. The 100% anchor was used for participants in the self-set goal group to induce choosing a difficult goal. We made a number of changes in Experiment 2, which are described below.

First, we modified the decision task and the measure used to assess escalation to create a robust setting that allowed us to replicate and extend Experiment 1. One change introduced in Experiment 2 was the level of investment made in Phase 1. While there are studies that have indicated that a small amount of prior investment can lead to escalation of commitment (e.g., Brockner et al., 1979; O'neill, 1986), we decided to more than double the task duration (from 1 to 2.5 min) in Phase 1 to create greater investments of time and effort. Further, for the letter-counting task, we used an article that was approximately 2.5 times longer than the article used in Experiment 1. Adopting a longer article helped ensure that the difficulty of the task was comparable to the task used in Experiment 1 (i.e., more time is given for a longer article). The article used in Experiment 2 had 582 words and contained 237 a’s (ESM 1). The percentage of a’s that a participant set or was assigned to identify in the article constituted the goal.

A second change involved the measure used for escalation of commitment. While escalation was previously measured by the percentage of time allocated to the original task, in Experiment 2, we employed an alternative measure that is also well-established in the escalation literature: namely, the willingness to continue a failing course of action (Conlon & Garland, 1993; Wong et al., 2006). We used two questions: (1) the probability of continuing the task from 0% to 100% (0 = absolutely no, 50 = neutral, and 100 = absolutely yes) and (2) the decision on whether to continue working on the task on a 10-point rating scale. The second question was later rescaled to a 100-point scale and used together with the first question to create a linear composite score.

**Figure 3:**
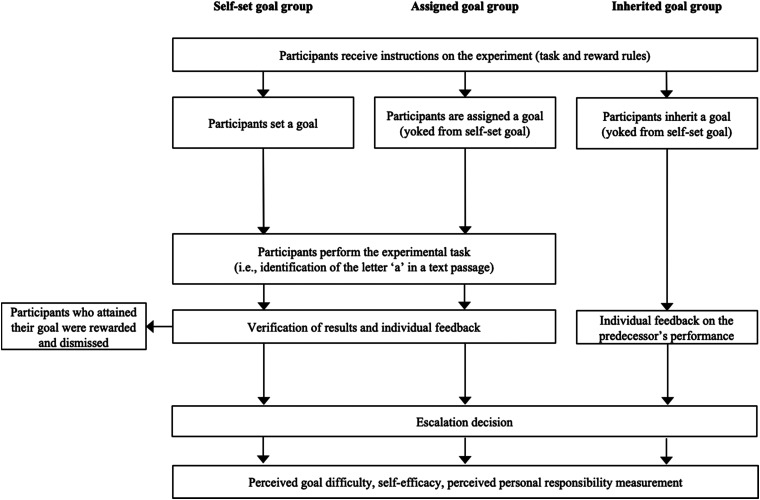
Experimental procedure (Experiment 2).

A third change was that we included a measure for perceived personal responsibility. We measured perceived personal responsibility using two questions: (1) I would feel responsible if I failed to achieve this goal, and (2) it is my responsibility to meet this goal. Finally, we measured perceived goal difficulty, self-efficacy, and personal responsibility at the end of the experiment (i.e., after the escalation decision). We did this to avoid any possible effect that completing these items might have on our ultimate dependent variable (i.e., escalation). We should note that self-efficacy and personal responsibility were measured after participants in the self-set and assigned goal conditions performed the task. For participants in the inherited goals condition, these were measured after they were informed about the predecessor’s goal and performance. This timing of measurement enabled us to control for self-efficacy and personal responsibility differences that may have existed across the experimental groups due to the experience involved in performing the task. Besides these changes, the experimental procedure was identical to Experiment 1. See Figure 3 for a graphical representation of the experimental procedure used in Experiment 2.

### Results

A triad of three participants sharing the same goal (a participant in the self-set goal condition, a participant in the assigned goal condition, and a participant in the inherited goal condition) became our unit of analysis. A total of four participants attained their goals in Phase 1 and were excluded from further analysis. Only those triads in which neither the participants in the self-set goal group nor those in assigned goal group attained their goal were included in the analysis. On this basis, 30 triads were retained for analysis.

**Figure 4:**
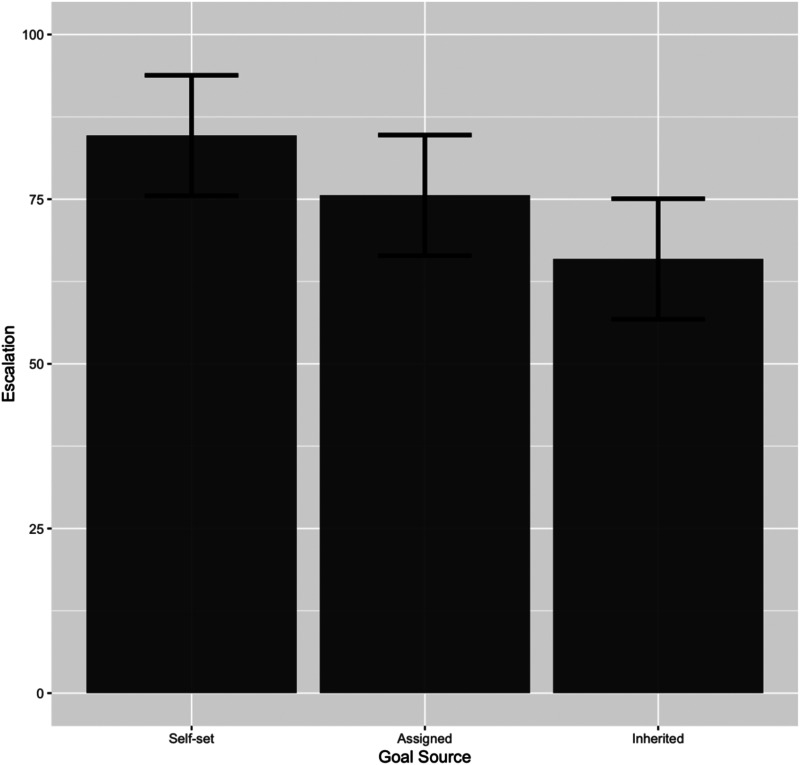
95% CIs of escalation decision (Experiment 2).

The mean goal set by the participants in the self-set goal group was 74.97 (*SD* = 20.40). Our yoked design ensured that all three treatment groups had identical goals. The dependent variable was the willingness to continue working on the previous task. Participants in the self-set goal group showed the greatest willingness to continue (*M* = 84.65, *SD* = 22.89), followed by those in the assigned goal group (*M* = 75.58, *SD* = 23.92), and those in the inherited goal group (*M* = 65.92, *SD* = 30.28). This pattern was consistent with the results of Experiment 1. 95% Confidence intervals (CI) calculated based on repeated-measure error terms (Loftus & Masson, 1994) for each experimental group and are shown in Figure 4.

Next, we conducted a repeated measures regression analysis with a least-squares dummy variable approach as before in Experiment 1 to test the relationship between goal source and escalation. First, in Model 1, the dummy variables were entered into the regression to predict the mean willingness to continue (DV). Second, in Model 2, self-efficacy and perceived personal responsibility were entered into the regression to control for between-subject differences in these factors. Third, in Model 3, the goal source was entered into the regression. The *F*-change in Model 3 was found to be significant (∆*F* = 10.47, *p* < .01), indicating that the goal source affected escalation controlling for individual differences in a triad, self-efficacy, and perceived personal responsibility.

Finally, we conducted a between-subjects ANCOVA with a post hoc analysis (Tukey HSD). Self-efficacy and perceived personal responsibility were entered as covariates. The results suggested a significant main effect of goal source on the willingness to continue [*F*(2,82) = 5.17, *p* < .01, η_*p*_^2^ = .11]. In contrast, personal responsibility, posited as a potential alternative explanation, was found to have no significant effect on the dependent variable (*p* = .71). A post hoc analysis (Tukey HSD) showed that willingness to continue was significantly higher in the self-set goal group than in the inherited goal group (*p* < .05). The findings of Experiment 2 suggest that there exists a relationship between goal source and escalation of commitment and that the observed effect is not due to differing levels of perceived personal responsibility induced by the different types of the goal source. While our manipulation of the goal source was similar to the way in which personal responsibility was manipulated in prior escalation studies (e.g., Staw, 1976), our findings suggest that (1) there is a cognitive effect introduced by the involvement in setting a goal, as opposed to merely inheriting a goal that someone else failed to achieve, and (2) the differences we observe from the goal source are not the result of differences in perceived personal responsibility.

## Discussion

Escalation of commitment is a common phenomenon that can adversely affect decision-making across a wide variety of contexts. While prior research has illuminated important aspects of the escalation phenomenon, the literature remains silent on how goal source shapes escalation behavior. In this research, we integrate a GST perspective to shed new light on the relationship between goal source and escalation behavior.

Our research contributes to the literature by providing new insights regarding the relationship between goal source and escalation of commitment. First, our work represents the first empirical study to systematically investigate the relationship between goal source and escalation. While there are a couple of studies (e.g., Lee et al., 2012, 2015) that use GST to examine escalation and initial goal setting, these studies have only examined goal difficulty and goal specificity. Our study provides a different perspective on initial goals and escalation by examining the goal source. Moreover, while prior research on GST has investigated the difference between self-set goals and assigned goals and their impact on task performance and goal commitment (e.g., Erez et al., 1990), inherited goals have not been discussed in the literature. Inherited goals, however, are important in the escalation context. Escalation research suggests that replacing the decision maker is effective for inducing de-escalation (Mähring et al., 2008), and one explanation for this may be that the new decision maker is less motivated when the goal is inherited. In sum, the main contribution of our work is to conceptualize inherited goals by marrying GST with escalation of commitment and empirically showing that such goals can help limit escalation of commitment relative to self-set goals.

Second, while one of the most prominent explanations of escalation of commitment is personal responsibility from self-justification theory (Staw, 1976), the results from Experiment 2 demonstrate that personal responsibility induced by setting one’s own goal is not the factor driving escalation; self-justification does not appear to be the reason for individuals with self-set goals to exhibit a greater willingness to continue. GST is a motivation theory which posits that the goal source influences one’s effort toward attaining a goal (Latham et al., 1988). Rather than triggering the need for self-justification by inducing a greater sense of personal responsibility, our research suggests that self-set goals are more likely to cause individuals to continue to pursue a failing course of action through goal achievement motivation.

Lastly, while we acknowledge that goal setting is an effective tool in producing greater effort, we wish to draw attention to the dark side of goal setting. In their seminal book, Locke and Latham (1984) pointed out several potential dangers of goal setting. Overall, our research provides additional evidence concerning the negative implications of goal setting, suggesting that under certain circumstances self-set goals can engender greater escalation of commitment relative to inherited goals.

### Limitations and Directions for Future Research

As with any research, there are limitations that must be noted. First, there is the issue of external validity. The experimental task in our studies involved identifying the occurrences of a particular letter in a small passage of text, and thus, the ability to generalize from our research to the organizational context is necessarily somewhat limited. However, laboratory experiments are still useful as they provide high levels of internal validity (Tybout et al., 2001). Our experimental task is more advantageous than the typical role-playing experiment used in escalation studies (e.g., Conlon & Garland, 1993; Staw, 1976; Wong et al., 2006) as it allows participants to become engaged in a task with a reward based on their performance, thereby enhancing the psychological realism of our experiment (Brockner et al., 1979). Thus, despite the limitation of external validity, we believe that our approach was robust and that the results hold important implications for research. Still, one direction for future research would be to determine the extent to which goal setting influences escalation of commitment in various field settings.

Second, participants spend a relatively small amount of time on the task before making an additional commitment decision. This investment of time represents a small fraction of what might be typically experienced in field settings. However, a small investment of time should also limit the tendency to engage in escalation, thus reducing the chance of finding statistically significant effects. Thus, our experimental context can be said to provide a conservative test of our hypothesis. Moreover, there are other escalation studies in settings that involved only a small amount of prior investment, such as waiting for a bus (e.g., Brockner et al., 1979) or participating in a one-dollar auction (e.g., O'neill, 1986). Nevertheless, future studies should investigate the effects of higher levels of investment in a task.

Third, we did not assess whether or not subjects perceived our experimental task or incentives associated with goal attainment to be meaningful.

Fourth, we did not consider goals that were set participatively (Locke & Latham, 2006). Rather, we conceptualized three distinct goal sources that could appear in escalation situations that involve individual decision making. The escalation literature primarily focuses on decisions to continue a failing course of action at the individual level, yet some studies suggest that escalation can occur at the group level (e.g., Whyte, 1993). Participating in setting goals jointly can lead to greater acceptance of the goal by group members, thereby increasing task performance (Erez et al., 1985). Thus, another interesting avenue for future research would be to investigate the impact of jointly set goals in escalation situations that involve group decision making.

Fifth, in creating an inherited goal source condition, it was necessary to have participants not perform the task in Phase 1. However, this may lead to a possible confound relating to amount of experience with the task relative to the assigned goal and self-set goal source conditions. We measured self-efficacy and perceived personal responsibility after participants performed the task (self-set and assigned goals) or received information on the predecessor’s goal and performance (inherited goals). This was because we felt that performing the task in Phase 1 may boost one’s self-efficacy or perceived personal responsibility. Therefore, including these two factors as covariates helped us to control for potential effects that may arise from performing the task versus not performing the task. Nonetheless, we suggest that further research is warranted to examine and tease out the effects associated with task experience in understanding the role of inherited goals (vs. self-set or assigned goals) in escalation of commitment.

Finally, since this was an initial effort to conceptualize and empirically test the effect of goal source on escalation, we did not examine any boundary conditions or moderating effects. Clearly, there may be some important moderating factors to consider. For example, goal commitment may serve as a moderator for the relationship between goal source and escalation. In addition, in our research, personal responsibility was not explicitly manipulated as in some previous escalation studies. It may be worthwhile to explicitly manipulate personality responsibility and examine how it may moderate the relationship between goal source and escalation. Further, while self-efficacy was used as a covariate in our research, its potential moderating effect can be examined in future research.

### Conclusion

In conclusion, the goal source can have significant implications with respect to individuals’ decision of whether or not to continue a previous course of action that has not produced the desired outcome. In this study, we conceptualize distinct goal sources in escalation situations and find that individuals with self-set goals are more willing to continue a troubled course of action than those with inherited goals, but this is not necessarily because of perceived personal responsibility. Our research underscores the strong connection between the source of the initial goal and escalation behavior by drawing upon GST, thus, offering new insights into the escalation phenomenon.

## Electronic Supplementary Materials

The electronic supplementary materials are available with the online version of the article at https://doi.org/10.1027/1618-3169/a000627

**ESM 1.** Instructions and setup for experiments.

## Footnotes

^1^
In this research, we study escalation of commitment to a failing course of action (or a previously chosen course of action that failed to generate the desired outcome). Escalation of commitment is different from what is commonly known as goal commitment in GST.

^2^
In GST, self-set and participatively set goals are conceptualized as two distinct types of goal source. However, empirically GST research has found that there are no meaningful differences in terms of their effects on performance (Locke & Latham, 2012). Therefore, in this research, we focus only on self-set goals.

## Notes

**Publication Ethics:** Informed consent was obtained from all participants included in the study. All procedures performed in studies involving human participants were in accordance with the ethical standards of the research ethics committee of Georgia State University's Institutional Review Board.

**Authorship:** Jong Seok Lee, Conceptualization, research design, data collection and analysis, writing – original draft. Mark Keil, Conceptualization, research design, writing – review & editing. Kin Fai Ellick Wong, Conceptualization, research design, writing – review & editing. Hyung Koo Lee, Writing – review & editing. All authors approved the final version of the article.

**Open Science:** To the best of my ability and knowledge, I have provided all original materials and clear references to all other materials via a stable online repository. The article includes a DOI generated by a public data repository pointing to the raw data underlying the findings reported in the article.

Open Data: Data are available at https://osf.io/u8vhq/ (Lee, 2024).

Open Materials: The materials for this study are available at https://osf.io/u8vhq/ (Lee, 2024).

Pre-registration: My manuscript contains no experiment with a completely executed preregistration.
