# Supplementary material for: The Role of Goal Source in Escalation of Commitment
Source: Exp Psychol. 2024 Dec 12;71(4):202–13. doi: 10.1027/1618-3169/a000627 (PMC11770365; doi:10.1027/1618-3169/a000627)
Supplement: Supplementary file 1 [file zea_71_4_202_esm1.pdf]

https://doi.org/10.1027/1618-3169/a000627

Electronic Supplementary Materials

Instructions and Setup for Experiment 1 (Self-Set and Assigned Goal Groups)

Instructions

The task that follows is part of a study that examines human performance. The task involves reading a passage of text and **circling the occurrences of the letter "a" in both upper and lower case form**. You will be given a goal or asked to choose one for yourself. **The goal will correspond to the percent of a's that you will identify in the passage of text.** You will be rewarded for your performance based on your ability to meet the goal. **Your reward will be in direct proportion to the percentage of total a's identified, provided that you meet the goal, but there will be no additional reward for exceeding the goal.** Rewards will be set at the rate of 10 cents for every percent of a's identified in the passage. In other words, if your goal is to identify 10% of the a's in the passage, you will receive $1 if you meet or exceed this goal. If, on the other hand, your goal is to identify 90% of the a's in the passage, you will receive $9 if you meet or exceed this goal. **If, however, you do not meet the goal, you will receive NOTHING, no matter how close you come to reaching it. There is no benefit to exceeding your goal, as there will be no additional monetary reward for exceeding the goal.** No rewards WILL be given for guessing (i.e., you must circle the a's that you identify). Please read all instructions carefully and respond to the questions below before starting the task. Do NOT skip ahead.

Set a goal for the percentage of 'a' letters in the passage that you think you can identify (by circling) in 1 minute, for example 100%. Record your goal below:

- My goal is to be able to identify and circle _______% of the "a" letters in 1 minute.

+++ Self-set goal+++

- Your goal is to identify and circle XX% of all 'a' letters in the article in 1 minute.

+++Assigned goal+++

Note. XX -> each subject was given a number based on the yoked design

https://doi.org/10.1027/1618-3169/a000627

You now have 1 minute to identify and circle the a's (both upper and lower case) in the paragraph below.

"Twitter has modified the terms of service that govern the proper user of the microblogging and social-networking site to state unequivocally that messages posted belong to their authors and not to the company. "Twitter is allowed to 'use, copy, reproduce, process, adapt, modify, publish, transmit, display and distribute' your tweets because that's what we do. However, they are your tweets and they belong to you," wrote Twitter co-founder Biz Stone in a blog post Thursday announcing the modifications. There has been controversy over the question of who owns the messages, photos, videos and other material that people post to social media and social-networking sites like Twitter, Facebook, MySpace and YouTube. For example, Google and Facebook got into hot water when critics complained about what they perceived as terms of service that claimed ownership of the data end users store in Google Apps and Facebook profiles. The revised Twitter terms also state that end users allow Twitter to make posted messages available to external applications that use the Twitter API (application programming interface). However, Twitter is still hammering out a set of guidelines for developers on the proper use of the API. The API guidelines are still in draft form and require that developers identify the authors of "tweets," maintain the integrity of the text and obtain permission to send messages on end users' behalf or turn their message into a commercial product, like a poster. Twitter is also keeping mum on details about the display of advertising on the site, an issue of much discussion among pundits who follow the company and have raised questions about how it will generate advertising to sustain its business. Twitter welcomes feedback on its terms of service and will revise them as it deems necessary."

https://doi.org/10.1027/1618-3169/a000627

In the space below, record the total number of “a’s” you identified and circled:

____

After you have recorded the number of a’s circled, please take your paper to the instructor and s/he will give you feedback on your performance.

https://doi.org/10.1027/1618-3169/a000627

Phase 2

You now have the opportunity to work for **2 additional minutes**. **You may allocate your time in any proportion you like between two tasks.** As before, you will be rewarded based on your performance. **The first task is to continue identifying and circling a's with the aim of meeting your previous goal.** If you choose this task and the cumulative performance (across Phase 1 and Phase 2) meets or exceeds your previous goal, you will win the reward under the same rules as before. **The second task involves setting a new goal and identifying and circling the number of i's in the same passage of text.** If you are able to meet or exceed your new goal for this task, you will receive a **reward in direct proportion to the percentage of total i's identified** under the same rules as before (i.e., getting the rewards if you meet or exceed the goal or no rewards if you do not meet the goal). You need to decide how to allocate your 2 minutes across the two tasks in any proportion you like. If you can meet or exceed your goals on both tasks, you can receive two rewards.

Be aware that you have already put 1 minute worth of effort on the original task and have achieved some progress. This progress will have no value, unless you choose to build upon it and are able to achieve the goal in the coming opportunity (i.e., Phase 2). **If you achieve both goals you will receive rewards for both, but if you don't achieve a goal, you will earn NOTHING for that task, no matter how close you come toward reaching your goal.** Please indicate what percentage of the 2 minutes you would choose to work on the two tasks. The sum should add to 100%.

---

1. I would choose to spend ___ % of my time on the original task.

1-1. I believe that my Phase 1 goal is achievable in Phase 2 ___YES ___No

---

2. I would choose to spend ___% of my time on the new task. If you choose to spend any

---

https://doi.org/10.1027/1618-3169/a000627

time on the new task, you must state your goal below:

My goal is to be able to identify and circle ____% of the “i” letters in the fraction of time allocated to this task.

https://doi.org/10.1027/1618-3169/a000627

Instructions and Setup for Experiment 1 (Inherited Goal Group)

Instructions

The task that follows is part of a study that examines human performance. The task involves reading a passage of text and **circling the occurrences of the letter "a" in both upper and lower case form**. **You have been brought in to replace another individual who had previously set a goal of identifying and circling XX% of all 'a' letters in 1 minute. You had nothing to do with the setting of his/her goal.** Your predecessor failed to achieve the XX% goal and was only able to identify XX% of all 'a' letters.

Now, you will be given **2 minutes** and **you may allocate your time in any proportion you like between two tasks**. **The first task is to continue identifying and circling a's with the aim of meeting your predecessor's goal**. If you choose this task and the cumulative performance (across yours and your predecessor's) meets or exceeds the previous goal, you will win a reward in direct proportion to the percentage of total a's identified, as explained in the award rules given below. **The second task involves setting a new goal and identifying and circling the number of i's in the same passage of text**. If you are able to meet or exceed your new goal for this task, you will receive a **reward in direct proportion to the percentage of total i's identified**. You need to decide how to allocate your 2 minutes across the two tasks in any proportion you like. If you can meet or exceed your goals on both tasks, you can receive two rewards.

Be aware that your predecessor had already put 1 minute worth of effort on the original task and had achieved some progress. This progress will have no value, unless you choose to build upon it and are able to achieve the goal in the coming opportunity. **If you achieve both goals you will receive rewards for both, but if you don't achieve a goal, you will earn NOTHING for that task, no matter how close you come toward reaching your goal**.

Award Rules

Your reward will be in direct proportion to the percentage of total a's or i's identified, provided that you meet the goal, but there will be no additional reward for exceeding the goal. Rewards will be set at the rate of 10 cents for every percent of a's or i's identified in the

passage. In other words, if your goal is to identify 10% of the a's in the passage, you will receive $1 if you meet or exceed this goal. If, on the other hand, your goal is to identify 90% of the a's in the passage, you will receive $9 if you meet or exceed this goal. **If, however, you do not meet the goal, you will receive NOTHING, no matter how close you come to reaching it.**

Note. XX -> each subject was given a goal and a performance based on the yoked design

https://doi.org/10.1027/1618-3169/a000627

Please indicate what percentage of the 2 minutes you would choose to work on the two tasks. The sum should add to 100%.

---

1. I would choose to spend ___ % of my time on the original task initiated by my predecessor.

1-1. I believe that the goal set by my predecessor is achievable in Phase 2 ___YES ___No

---

2. I would choose to spend ___% of my time on the new task. If you choose to spend any time on the new task, you must state your goal below:

My goal is to be able to identify and circle ____% of the “i” letters in the fraction of time allocated to this task.

---

https://doi.org/10.1027/1618-3169/a000627

Instruments and Setup Used in Experiment 2

Instructions

The task that follows is part of a study that examines human performance. The task involves reading a passage of text and **circling the occurrences of the letter "a" in both upper and lower case form in 2.5 minutes**. {[You will be given a goal] [You will be asked to choose a goal for yourself}. **The goal will correspond to the percent of a's that you will identify in the passage of text.** You will be rewarded for your performance based on your ability to meet the goal. {**[Proportional reward] Your reward will be in direct proportion to the percentage of total a's identified, provided that you meet the goal, but there will be no additional reward for exceeding the goal.** Rewards will be set at the rate of 10 cents for every percent of a's identified in the passage. In other words, if your goal is to identify 10% of the a's in the passage, you will receive $1 if you meet or exceed this goal. If, on the other hand, your goal is to identify 90% of the a's in the passage, you will receive $9 if you meet or exceed this goal. **[Flat reward] You will receive a $5 reward if you meet or exceed your goal, but there will be no additional reward for exceeding the goal}**. **If, however, you do not meet the goal, you will receive NOTHING, no matter how close you come to reaching it.** No rewards will be given for guessing (i.e., you must circle the a's that you identify). Please read all instructions carefully and respond to the questions below before starting the task. Do NOT skip ahead.

• My goal is to be able to identify and circle ______% of the "a" letters in 2.5 minutes

+++ Self-set goal+++

• Your goal is to identify and circle XX % of all 'a' letters in the article in 2.5 minutes.

+++ Self-set goal+++

Note. XX -> each subject was given a number based on the yoked design

https://doi.org/10.1027/1618-3169/a000627

The passage of text appears below.

“Democrats and Republicans are dug in like soldiers at Verdun over what to do about the sputtering U.S. economy. Exhausted by the political stalemate, they've been reduced to magical thinking, hoping that things will eventually get better by themselves. But time isn't on America's side. The country is suffering its highest average duration of unemployment since at least 1948. "The longer this goes on, the greater the danger that the cyclical downturn becomes structural. People and things that lie idle start to lose their productive value. Then you're into all sorts of troubles," says Karen Ward, senior global economist at HSBC Holdings (HBC) in London. It may finally be time for Americans to consider ideas from a place that they don't usually look to for inspiration: the rest of the planet. The U.S.'s economic predicament does present some unique dilemmas. The Obama Administration has already pushed hard on the levers that countries usually use to get out of a slump, to little discernible effect. Short-term interest rates are near zero and fiscal stimulus is aggressive, judging from a budget deficit of about 10 percent of gross domestic product. David Rosenberg, chief economist at Gluskin Sheff & Associates, a Toronto-based wealth-management firm, says of the U.S.: "We're basically in uncharted territory." Maybe so. But there are guideposts that can help point the way out. The U.S., after all, is not the first country to wrestle with how to restart growth despite budget deficits that constrain big-spending solutions. The challenge is how to apply lessons from other countries to shore up American weaknesses, without sacrificing the strengths that make the U.S., for all its troubles, the world's biggest economy. To prod the conversation forward, *Bloomberg Businessweek* scanned the world and found innovative economic ideas in countries as diverse as Germany, Brazil, Singapore, and Thailand that are applicable to America's

https://doi.org/10.1027/1618-3169/a000627

mess. The focus was on short-term solutions, but since there aren't a whole lot of miracle fixes to be had, we also considered some longer-term reforms that create a better environment for years of sustainable growth. There's no guarantee that all of these ideas would work in an American context. But it's clear that some fresh, non-ideological thinking is needed. Says Dow Chemical (DOW) Chief Executive Officer Andrew N. Liveris, a Greek-Australian-American and author of the book *Make It in America: The Case for Re-Inventing the Economy*: "People in the U.S. confuse big government and small government as the only two models. What we need is smart government." By that he means government that puts business objectives ahead of politics. "Countries are competing like companies more and more," says Liveris, "In the U.S., we haven't caught up." Here are nine ideas from the rest of the world to get America back in the race. Germany has one of the lowest homeownership rates among wealthy nations—around 46 percent, vs. two-thirds in the U.S.—and also one of the most stable housing markets. Prices of owner-occupied housing in Germany are up 9 percent since 2003, according to the Association of German Pfandbrief Banks. What's the German formula? Housing is less vulnerable to booms and busts because only highly qualified buyers can get a mortgage. Down payments are usually at least 20 percent, often 40 percent. Mortgage interest is not tax-deductible, as it is in the U.S., which also discourages excessive leverage. Germans are justly proud of their Pfandbrief, an ultrasafe bond whose collateral is a set of standardized mortgages whose loan-to-value ratio can't exceed 60 percent.

In the space below, record the total number of “a’s” you identified and circled:

____

https://doi.org/10.1027/1618-3169/a000627

After you have recorded the number of a's circled, please take your paper to the instructor and s/he will give you feedback on your performance.

https://doi.org/10.1027/1618-3169/a000627

Phase 2

You now have the opportunity to work for another **2.5 minutes**. **You may continue to identify and circle a's with the aim of meeting the previous goal.** If you choose to continue and your cumulative performance (across Phase 1 and Phase 2) meets or exceeds the previous goal, you will win the reward under the same rules as before.

Be aware that you have already put 2.5 minutes worth of effort on the task and have achieved some progress. This progress will have no value, unless you choose to build upon it and are able to achieve the goal in the coming opportunity (i.e., Phase 2). **If you achieve the previous goal, you will receive the reward, but if you don't achieve the previous goal, you will earn NOTHING for that task, no matter how close you come toward reaching the goal.**

Please indicate your willingness to continue to pursue the the goal below as a percentage probability from 0 to 100% (0 = absolutely no, 50 = neutral, 100 = absolutely yes).

I would like to continue identifying and circling a' with the aim of meeting the previous goal	A percentage probability from 0 to 100% **(0 = absolutely no, 50 = neutral, 100 = absolutely yes)**<br><br>__________ %

Further, please indicate your final decision of whether or not to continue working this database project.

I would continue working on this task to achieve the previous goal:

Absolutely disagree	Strongly disagree	Disagree	Somewhat disagree	Slightly disagree	Slightly agree	Somewhat agree	Agree	Strongly agree	Absolutely agree
|	|	|	|	|	|	|	|	|	|
□	□	□	□	□	□	□	□	□	□

https://doi.org/10.1027/1618-3169/a000627

Instructions and Setup for Experiment 2 (Inherited Goal Group)

Instructions

The task that follows is part of a study that examines human performance. The task involves reading a passage of text and **circling the occurrences of the letter "a" in both upper and lower case form**. **You have been brought in to replace another individual who had previously set a goal of identifying and circling XX% of all 'a' letters in 2.5 minutes. You had nothing to do with the setting of his/her goal.** Your predecessor was only able to identify XX %, thus having failed to achieve the goal, XX%.

Now, you will be given **2.5 minutes to continue identifying and circling a's with the aim of meeting your predecessor's goal**. If you choose this task and the cumulative performance (across yours and your predecessor's) meets or exceeds the previous goal, [you will win a reward in direct proportion to the percentage of total a's identified, as explained in the award rules given below/you will receive a $5 reward as explained in the award rules given below]. You need to decide whether or not you continue identifying and circling a's with the aim of meeting your predecessor's goal.

Be aware that your predecessor had already put 2.5 minutes worth of effort on the original task and had achieved some progress. This progress will have no value, unless you choose to build upon it and are able to achieve the goal in the coming opportunity (i.e., Phase 2). **If you achieve your predecessor's goal, you will receive a reward, but if you don't achieve the goal, you will earn NOTHING for that task, no matter how close you come toward reaching your predecessor's goal**.

https://doi.org/10.1027/1618-3169/a000627

Award Rules

[Your reward will be in direct proportion to the percentage of total a's identified, provided that you meet the goal, but there will be no additional reward for exceeding the goal. Rewards will be set at the rate of 10 cents for every percent of a's identified in the passage. In other words, if your goal is to identify 10% of the a's in the passage, you will receive $1 if you meet or exceed this goal. If, on the other hand, your goal is to identify 90% of the a's in the passage, you will receive $9 if you meet or exceed this goal.] [You will receive a $5 reward if you meet or exceed your goal, but there will be no additional reward for exceeding the goal.] **If, however, you do not meet the goal, you will receive NOTHING, no matter how close you come to reaching it.**

Note. XX -> each subject was given a goal and a performance based on the yoked design
